# A machine learning model for predicting the risk of diabetic nephropathy in individuals with type 2 diabetes mellitus

**DOI:** 10.3389/fendo.2025.1587932

**Published:** 2025-10-15

**Authors:** Tingting Li, Jinbo Chen, Xin Zhang, Kaiwen Wang, Xuesen Zhao, Yi Cao, Zhen Xu, Shiyue Wang, Peng Su, Xiaoyan He, Yang Yang, Xiaolu Cao, Xiaohua Liang, Dong Ma

**Affiliations:** 1 Department of Biochemistry and Molecular Biology, Key Laboratory of Neural and Vascular Biology, Ministry of Education, Shijiazhuang, Hebei, China; 2 Hebei Key Laboratory of Cardiovascular Homeostasis and Aging, Hebei Medical University, Shijiazhuang, Hebei, China; 3 School of Public Health, North China University of Science and Technology, Tangshan, China; 4 Department of General Medicine, Shijiazhuang Second Hospital, Shijiazhuang, China; 5 School of Medicine, Hebei University of Engineering, Handan, China; 6 College of Public Health, Zhengzhou University, Zhengzhou, China; 7 Diabetic Ophthalmology Department, Hebei Eye Hospital, Xingtai, China

**Keywords:** type 2 diabetes mellitus, diabetic kidney disease, machine learning, prediction model, predictive value

## Abstract

**Introduction:**

Diabetic kidney disease (DKD) represents the predominant form of chronic kidney disease (CKD) linked with diabetes mellitus. The application of artificial intelligence holds promise for delaying renal deterioration and decreasing treatment expenses by facilitating early detection and intervention. This is contingent upon the development of an efficient and user-friendly model for predicting DKD risk in diabetic individuals. In this study, leveraging extensive clinical datasets, we sought to develop and validate a predictive model employing machine learning techniques to assess the risk of DKD in patients with type 2 diabetes mellitus (T2DM).

**Research design and methods:**

We conducted a retrospective collection of clinical data from 10,057 patients diagnosed with T2DM at Shijiazhuang Second Hospital. A random selection of 15% of these patients (n=1,508) was utilized for external validation. The remaining 8,549 patients were divided into a training set (*n* = 5,985) and a validation set (*n* = 2,564) using a simple random sampling method in a 7:3 ratio. Subsequently, we employed LASSO regression to identify variables significantly associated with DKD in T2DM patients. These variables were incorporated into eight distinct predictive models: Logistic Regression (LR), Random Forest (RF), Support Vector Machine (SVM), Gaussian Naive Bayes (GNB), KNeighbors Classifier (KNN), Gradient Boosting Classifier (GBM), AdaBoost Classifier (AdaBoost), and Extreme Gradient Boosting (XGBoost). The models’ predictive performance was assessed using metrics such as the area under the curve (AUC), accuracy, F1 score, and Brier score. Finally, we developed an online calculator to estimate DKD risk in T2DM patients.

**Results:**

Fifteen features—namely gender, age, systolic blood pressure (SBP), blood urea nitrogen (BUN), creatinine (Cr), BUN/Cr ratio, uric acid (UA), hemoglobin A1c (HbA_1c_), microalbuminuria, presence of diabetic retinopathy (DR), hypertension, coronary heart disease (CHD), history of cerebral infarction, family history of diabetes, and family history of CHD-associated with DKD were selected using LASSO regression. Among eight evaluated models, the XGBoost algorithm demonstrated superior performance on both training and validation datasets, with an AUCof 0.932 (95%*CI*: 0.926-0.938) and 0.930, (95%*CI*: 0.920-0.939), respectively. The model achieved an accuracy of 0.845 and 0.844, sensitivity of 0.834 and 0.850, specificity of 0.857 and 0.837, F1 score of 0.847 and 0.848, and a Brier score of 0.167 and 0.166, respectively. Decision curve analysis (DCA) further validated the superiority of the XGBoost model over other models across a range of clinically relevant risk thresholds, yielding the highest net benefits. Finally, an online predictive calculator for the occurrence of DKD was developed based on the XGBoost model, utilizing a cut-off value of 50.7%.

**Conclusions:**

The developed XGBoost model demonstrated optimal predictive accuracy for the occurrence of DKD in patients with T2DM. This model facilitated the construction of an online prediction calculator, offering an accessible and practical tool for both patients and clinicians.

## Introduction

Type 2 diabetes mellitus (T2DM) is the predominant form of diabetes, accounting for over 90% of diabetes cases. Diabetic kidney disease (DKD) is the most prevalent form of chronic kidney disease (CKD) associated with diabetes mellitus. In China, the prevalence of diabetes mellitus is approximately 170 million individuals (1), with 30% to 40% of these patients expected to develop DKD (2). Globally, DKD impacts 8% to 16% of the population’s health (3), and is characterized by a prolonged disease course, poor prognosis, and high treatment costs, imposing a significant burden on patients, families, and society. DKD is also a leading cause of end stage kidney disease (ESKD) (4, 5) and is now associated with a higher prevalence of cardiovascular diseases compared to other CKD patients (59.26% vs. 29.60%) (6). An international systematic review examining the prevalence and risk factors of DKD worldwide reported that the prevalence of DKD among T2DM patients ranges from 30% to 50% (7). Pan et al. (8) analyzed the burden of DKD in China from 1990 to 2019 and found that the increase in CKD cases is primarily attributed to the rising incidence of both T1DM and T2DM, with the number of prevalent T2DM cases with concomitant CKD being notably higher [57.4 (95%*CI*: 49.5-66.5) vs. 3,107.6 (95%*CI*: 2,815.2-3,390.9) million cases]. Consequently, a significant public health challenge lies in the precise and convenient prediction of high-risk diabetic kidney disease (DKD) in patients with diabetes. This early identification and intervention are anticipated to delay renal impairment and effectively reduce treatment costs.

There is a critical need for prognostic tools that are both easily interpretable and accurate, and that can be seamlessly integrated into clinical workflows. While certain blood-based biomarkers, such as plasma KIM-1 and TNF-α receptors, have shown correlation with the progression of DKD [like as plasma KIM-1 (9) and TNF-αreceptors (10)], the development of precise predictive models that incorporate patients’ electronic health records (EHR), including blood these biomarkers and other relevant factors remains limited. Machine learning, a vital component of artificial intelligence, is characterized by its ability to handle nonlinearity, complex interactions, and a greater number of variables influencing outcomes. This presents significant potential for enhancing the predictive capabilities of diseases models in clinical application. A growing body of literature indicates that several established predictive models, utilizing multifactor Logistic regression, BP neural networks, and LASSO regression, have been applied to screen risk factors for DKD complications in patients with T2DM (11, 12). However, a comparative analysis of the performance of these machine learning-based multi-predictive models remains unexplored. Consequently, this study aims to evaluate eight constructed DKD prediction models, to identify the most effective model for predicting the risk of DKD development in T2DM patients. To enhance the accessibility and utility of this model, we have developed an online calculator designed to assist clinicians in accurately stratifying risk and advising patients on the initial and progressive stages of DKD. Additionally, this tool aims to increase awareness of preventive measures in patients’ daily lives.

## Research design and methods

### Study participants

This retrospective study collected data from 10, 057 patients diagnosed with T2DM at the Second Hospital of Shijiazhuang City between December 2017 and December 2023. T2DM was defined according to the Guidelines for the Prevention and Treatment of T2DM in China (13) as follows: 1) T2DM was recorded in the medical billing; 2) the HbA_1c_ level was equal to or above 6.5% (NGSP); 3) the fasting plasma glucose level was equal to or above 126 mg/dL, except in an emergency room; 4) the postprandial plasma glucose level was equal to or above 200 mg/dL, except in an emergency room; 5) anti-diabetic medication was prescribed. In addition, the age of the diabetic patients was above 18 years. The exclusion criteria were as follows: 1) presence of concurrent chronic kidney disease (CKD) unrelated to diabetes; 2) coexistence of severe systemic diseases; 3) acute metabolic disorders; 4) incomplete demographic information or relevant laboratory indicators. This research was approved by the Ethics Committee of the Second Hospital of Shijiazhuang City (ethical approval number: NO. 191128). All private personal information was protected and removed during the analysis and publication process. Due to the retrospective nature of this study, written informed consent was not required.

### Definition of DKD

Focusing on one of the diabetic complications, concurrent DKD categorized all patients with T2DM into the DKD group (*n* = 5,162) and the non-DKD group (*n* = 4,895). The diagnostic criteria of DKD were as follows (14): 1) under conditions where diabetes is confirmed as the cause of renal damage as well as chronic kidney disease (CKD) was excluded; 2) albumin-to-creatinine ratio (UACR) ≥30 mg/g, urinary albumin excretion rate (UAER) ≥30 mg/24 h (or≥20 μg/min), and estimated glomerular filtration rate (eGFR) persistently < 60 ml·min^-1·^(1.73 m^2^) ^-1^ of three tests were conducted within a period of 3 to 6 months; 3) renal biopsy results consistent with pathological changes in DKD.

### Clinical data

First, we randomly selected 15% of the patients for external validation (*n* = 1,508) and used a simple random sampling method to divide the 8,549 patients into a training set (*n* = 5,985) and validation set (*n* = 2,564) in a ratio of 7:3. Clinical data of patients with T2DM collected through review of medical records were involved in four parts: 1) general information: gender, age, body mass index (BMI), systolic blood pressure (SBP), diastolic blood pressure (DBP), smoking history, alcohol consumption history, history of coronary heart disease, history of cerebral infarction, family history of hypertension, family history of diabetes, family history of coronary heart disease (CHD); 2) laboratory examination indicators: Triglycerides (TG), total cholesterol (TC), high-density lipoprotein (HDL), low-density lipoprotein (LDL), fasting blood glucose (FBG), glycated hemoglobin (HbA_1c_), high-sensitivity C-reactive protein (hs-CRP), albumin (Alb), white blood cell count (WBC), lymphocyte count (LYM), neutrophil count (NEUT), monocyte count (MONO), platelet count (PLT), platelet distribution width (PDW), large platelet ratio (P-LCR), D-dimer, blood urea nitrogen (BUN), creatinine (Cr), BUN/Cr, glucose (GLU), Apolipoprotein-A1/Apolipoprotein-B (APOA1/APOB), direct bilirubin (DBIL),indirect bilirubin (IBIL), microalbuminuria, α1-microglobulin (α1-MG), β2-microglobulin (β2-MG), uric acid (UA), aspartate transaminase (AST), alanine transaminase (ALT); 3) comorbidity status: diabetic retinopathy (DR), presence of hypertension, CHD, cerebral infarction, hypokalemia, hyperlipidemia.

### Statistical analysis

Continuous variables are presented as median (interquartile range), and categorical variables are expressed as the number of patients (%). The *t*-test or chi-square test was used to compare differences between the two groups. DKD occurrence in the training set was used as the dependent variable. Feature selection related to DKD was performed using least absolute shrinkage and selection operator (LASSO) regression. Based on these selected variables, eight distinct prediction models including: Logistic Regression (LR) model, Random Forest (RF) model, Support Vector Machine (SVM) model, Gaussian Naive Bayes (GNB) model, KNeighbors Classifier (KNN) model, Gradient Boosting Classifier (GBM) model, AdaBoost Classifier (AdaBoost) model, and Extreme Gradient Boosting (XGBoost) model were developed to achieve the idea predictive performance, which was further assessed by comparing the area under the receiver operating characteristic curve (AUC), accuracy, F1 score, and Brier score. Clinical utility metrics were evaluated using a decision curve analysis (DCA). After determining the best-performing model, the significant variables were visualized using xgb. plot and further interpretation of the XGBoost model using R Studio. Using the established XGBoost model, we calculated the area under the curve, accuracy, sensitivity, and specificity for predicting the occurrence of DKD in the external validation set. Lastly, the online XGBoost model via the Shiny package hosted on shinyapps.io, acting as a web-based predictor, was found to significantly drive the outcome, which conveniently and accurately estimates the risk of DKD in patients with T2DM. Statistical significance was set at *p* < 0.05. Analyses were performed using R version 4.4.2 and Python 3.13.2.

## Results

### Patient characteristics

**Figure 1:**
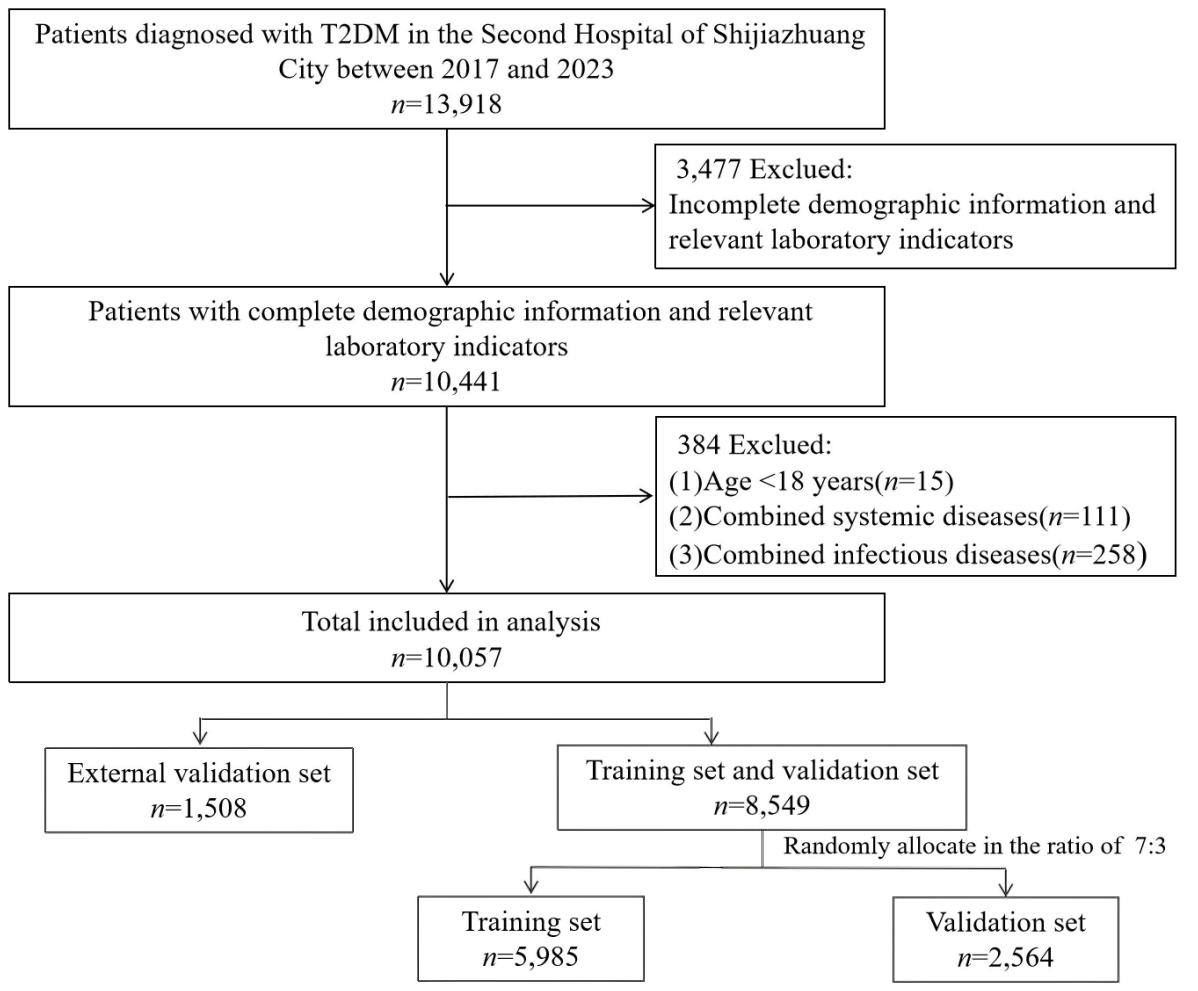
Flow chart of patient enrollment.

In total, 10,057 T2DM patients were enrolled in the present study based on the inclusion and exclusion criteria (**Figure 1**). **Table 1** shows patient characteristics according to the DKD complication accompanied by some significant differences in age, hs-CRP, IBIL, and history of cerebral infarction (all *P* < 0.05) observed between the training and validation sets.

**Table 1:** Baseline characteristics of the participants between training set and validation set.

Clinical Data	training set (*n* = 5,985)	validation set (*n* = 2,564)	*t* (*x*²) value	*P* value
Female [*n* (%)]	2,522(42.14)	1,105(43.10)	0.674^*^	0.411
Age,years	60.5 ± 12.5	59.6 ± 12.5	3.076	0.002
BMI, kg/m^2^	28.63 ± 38.20	27.39 ± 21.08	1.927	0.054
SBP, mmHg	136 ± 19	136 ± 19	0.873	0.383
DBP, mmHg	81 ± 12	81 ± 12	-0.396	0.692
Smoking [*n* (%)]	1,327(22.17)	593(23.13)	0.942^*^	0.332
Drinking [*n* (%)]	1,119(18.70)	518(20.20)	2.630^*^	0.105
FBG, mmol/L	19.28 ± 575.22	11.70 ± 27.18	0.666	0.505
hs-CRP, mg/L	18.53 ± 306.37	9.62 ± 34.67	2.216	0.027
WBC, ×10^9^/L	7.03 ± 22.35	6.59 ± 2.20	1.009	0.313
LYM, ×10^9^/L	2.07 ± 6.46	1.93 ± 2.21	1.008	0.313
NEUT, ×10^9^/L	4.50 ± 7.95	4.79 ± 25.95	-0.785	0.432
MONO, ×10^9^/L	0.49 ± 1.63	0.50 ± 2.00	-0.168	0.866
PLT, ×10^9^/L	227.09 ± 76.44	234.37 ± 309.84	-1.700	0.089
PDW, %	13.41 ± 5.64	13.39 ± 4.96	0.106	0.916
P-LCR, %	26.34 ± 27.60	26.06 ± 8.27	0.518	0.604
D-dimer, mg/L	0.51 ± 2.38	0.63 ± 3.69	-1.618	0.106
BUN, mmol/L	6.09 ± 7.37	5.85 ± 4.10	1.511	0.131
Cr, μmol/L	80.27 ± 95.06	78.52 ± 73.06	0.833	0.405
BUN/Cr	41.57 ± 54.13	42.51 ± 54.29	-0.730	0.465
UA, μmol/L	295.33 ± 98.82	297.47 ± 109.47	-0.885	0.376
GLU, g/L	14.15 ± 55.23	14.55 ± 65.13	-0.288	0.774
HbA_1c_, %	8.66 ± 2.12	8.61 ± 3.11	0.778	0.436
TG, mmol/L	2.64 ± 17.31	2.49 ± 10.41	0.408	0.683
TC, mmol/L	4.91 ± 11.27	4.74 ± 2.45	0.744	0.457
HDL, mmol/L	1.30 ± 3.75	1.27 ± 2.15	0.478	0.632
LDL, mmol/L	2.75 ± 5.64	3.06 ± 11.57	-1.652	0.099
APOA1/APOB	2.64 ± 21.49	2.11 ± 13.05	1.405	0.160
AST, U/L	22.54 ± 30.32	23.03 ± 34.86	-0.649	0.517
ALT, U/L	25.73 ± 34.13	27.74 ± 78.68	-1.248	0.212
DBIL, μmol/L	4.73 ± 8.37	4.91 ± 10.40	-0.879	0.379
IBIL, μmol/L	9.66 ± 4.82	9.95 ± 6.90	-2.167	0.030
Microalbuminuria, g/L	41.70 ± 8.95	48.83 ± 293.89	-1.228	0.220
α1-MG, mg/L	6.44 ± 92.03	4.62 ± 6.10	1.001	0.317
β2-MG, mg/L	9.56 ± 156.30	6.67 ± 110.91	0.848	0.397
ALB, g/L	24.12 ± 68.47	24.29 ± 45.90	-0.117	0.907
DR [*n* (%)]	2,243 (37.48)	923 (36.00)	1.683^*^	0.195
Hypertension [*n* (%)]	3,466 (57.91)	1,458 (56.86)	0.806^*^	0.369
CHD [*n* (%)]	2218 (37.06)	917 (35.76)	1.296^*^	0.255
Cerebral infarction [*n* (%)]	1,572 (26.27)	628 (24.49)	2.951^*^	0.086
Hypokalemia[*n* (%)]	172 (2.87)	64 (2.50)	0.954^*^	0.329
Hyperlipidemia[*n* (%)]	1,116 (18.65)	520 (20.28)	3.098^*^	0.078
History of coronary heart disease [*n* (%)]	1,681 (28.09)	695 (27.11)	0.860^*^	0.354
History of cerebral infarction [*n* (%)]	1,159 (19.37)	446 (17.39)	4.570^*^	0.033
Family history of hypertension [*n* (%)]	726 (12.13)	345(13.46)	2.877^*^	0.090
Family history of diabetes [*n* (%)]	2,116(35.36)	910(35.49)	0.015^*^	0.904
Family history of CHD [*n* (%)]	379(6.33)	157(6.12)	0.134^*^	0.715

^*^ is the x²value; 1 mmHg=0.133 kPa.

#### Identification of feature variables

**Figure 2:**
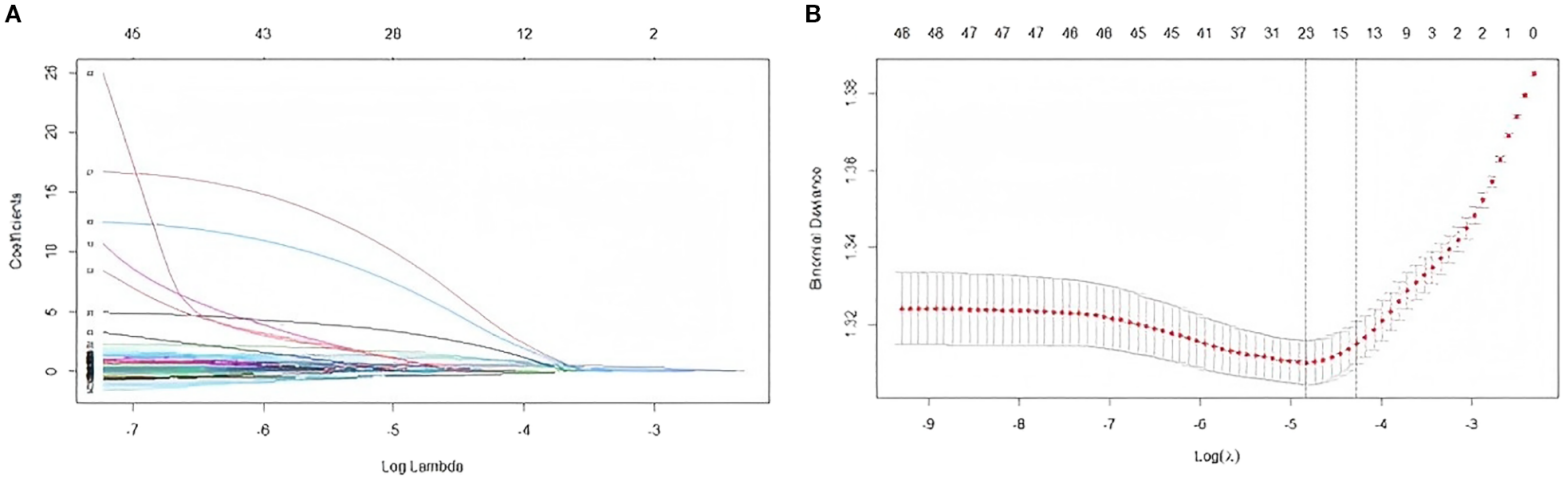
Identification of variables by LASSO regression. **(A)** Coefficient curves for the 47clinical features, **(B)** Selection of optimal variables through 10-fold cross-validation.

Through the variable assignment details shown in **Supplementary Table 1**, we applied LASSO regression using non-zero coefficients to further identify some strong variables to optimize the predictive model. With a 10-fold cross-validation for the optimal lambda value (lambda.1se=0.01397873), we ultimately selected 15 features relative to DKD, which included sex, age, SBP, BUN, Cr, BUN/Cr, UA, HbA_1c_, microalbuminuria, presence of DR, hypertension, CHD, history of cerebral infarction, family history of diabetes, and family history of CHD (**Figures 2A, B**).

#### Comparison of predictive models

**Figure 3:**
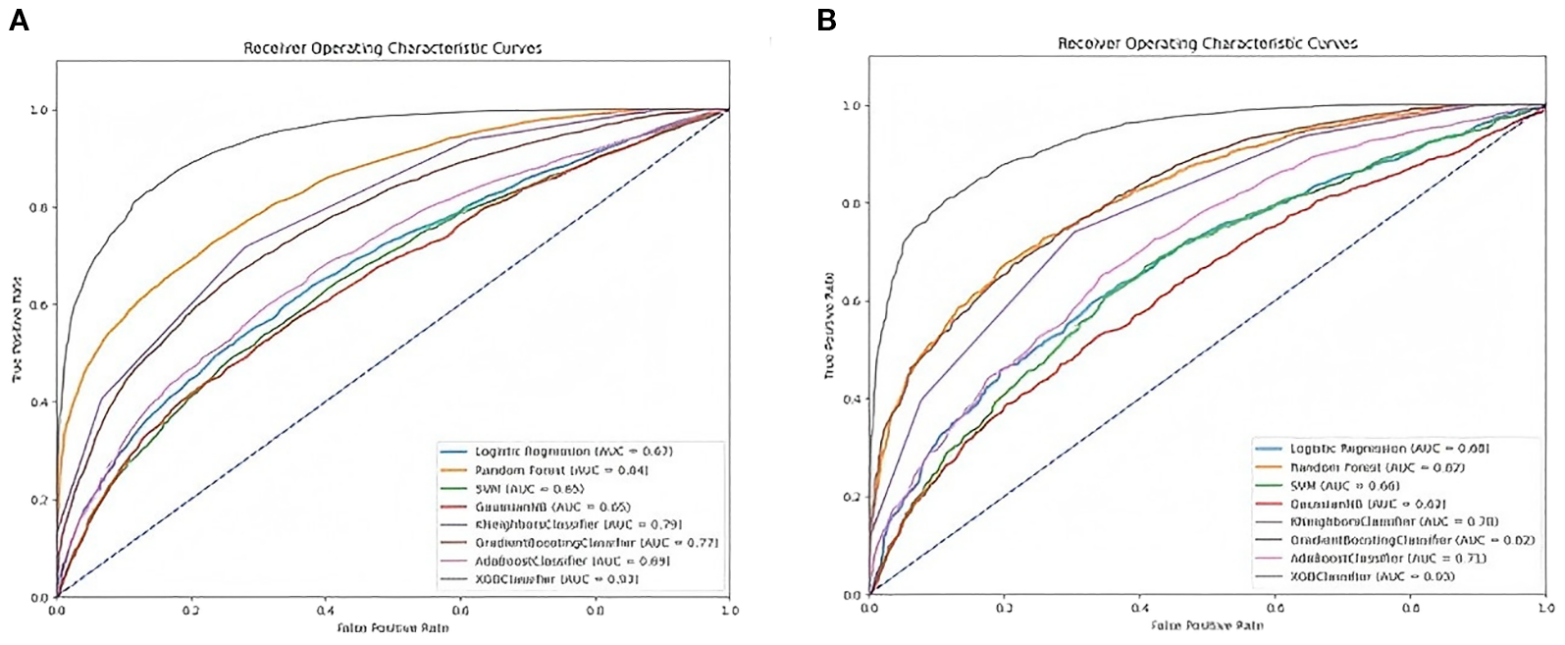
Receiver-operating characteristic curves for eight machine learning models. **(A)** Comparison of AUCs among the eight machine learning models in the training set, **(B)** Comparison of AUCs among the eight machine learning models in the validation set.

**Figure 4:**
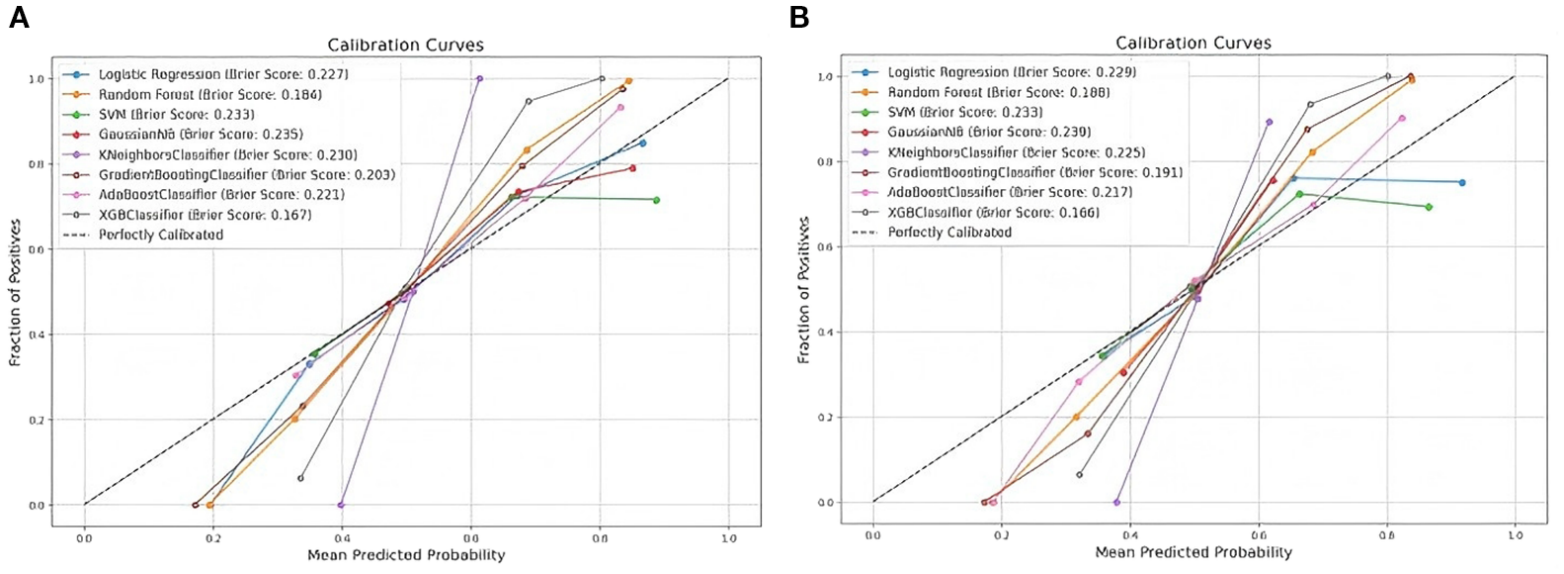
Calibration plots of the eight models. **(A)** Comparison of calibration plots among eight machine learning models in the training set and **(B)** comparison of calibration plots among eight machine learning models in the validation set.

We separately integrated the above 15 key variables into each of the eight machine learning models to compare the predictive ability of developing DKD risk in patients with T2DM. As shown in **Figure 3**, in the training set, using 10-fold cross-validation for discrimination, the mean AUC for the XGBoost model was the highest (0.932 95%*CI* (0.926-0.938), as well as; accuracy 0.845, sensitivity 0.834, specificity 0.857, and F1 score, 0.847 (**Figure 3A** and **Table 2**). Consistently, comparison among these models in the validation set showed that the XGBoost model also presented the best performance (AUC = 0.930, 95%*CI* (0.920-0.939), an accuracy of 0.844, a sensitivity of 0.850, a specificity of 0.837, and an F1 score of 0.848 (**Figure 3B** and **Table 3**). The calibration plots of the eight models show that XGBoost achieved better Brier scores (0.167 in the training set and 0.166 in the validation set) than the other models (**Figure 4**). This suggests that the XGBoost model is optimal for predicting the DKD risk in T2DM patients.

**Table 2:** Comparison of the performance metrics for eight models in the training set.

Model	AUC (95%*CI*)	Accuracy	Sensitivity	Specificity	F1 score	Cut-off value
LR	0.675 (0.662,0.688)	0.628	0.625	0.631	0.633	0.531
RF	0.839 (0.829,0.848)	0.743	0.699	0.790	0.737	0.487
SVM	0.653 (0.639,0.666)	0.611	0.551	0.674	0.593	0.545
GNB	0.646 (0.633,0.660)	0.531	0.126	0.959	0.216	0.159
KNN	0.791 (0.781,0.802)	0.718	0.717	0.720	0.723	0.600
GBM	0.767 (0.755,0.778)	0.696	0.688	0.704	0.699	0.530
AdaBoost	0.693 (0.679,0.706)	0.642	0.666	0.616	0.656	0.516
XGBoost	0.932 (0.926,0.938)	0.845	0.834	0.857	0.847	0.507

LR, Regression model; RF, Random Forest model; SVM, Support Vector Machine model; GNB, Gaussian Naive Bayes model; KNN, KNeighbors Classifier model; GBM, Gradient Boosting Classifier model; AdaBoost, AdaBoost Classifier model; XGBoost, Extreme Gradient Boosting model

**Table 3:** Comparison of the performance metrics for eight models in the validation set.

Model	AUC (95%*CI*)	Accuracy	Sensitivity	Specificity	F1 score	Cut-off value
LR	0.675 (0.653,0.695)	0.628	0.653	0.601	0.643	0.520
RF	0.817 (0.801,0.831)	0.734	0.732	0.736	0.738	0.523
SVM	0.661 (0.640,0.681)	0.618	0.568	0.670	0.604	0.482
GNB	0.624 (0.602,0.645)	0.587	0.685	0.484	0.630	0.610
KNN	0.782 (0.765,0.798)	0.719	0.739	0.698	0.729	0.600
GBM	0.821 (0.805,0.837)	0.728	0.752	0.704	0.739	0.547
AdaBoost	0.706 (0.686,0.727)	0.652	0.643	0.663	0.655	0.498
XGBoost	0.930 (0.920,0.939)	0.844	0.850	0.837	0.848	0.538

**Figure 5:**
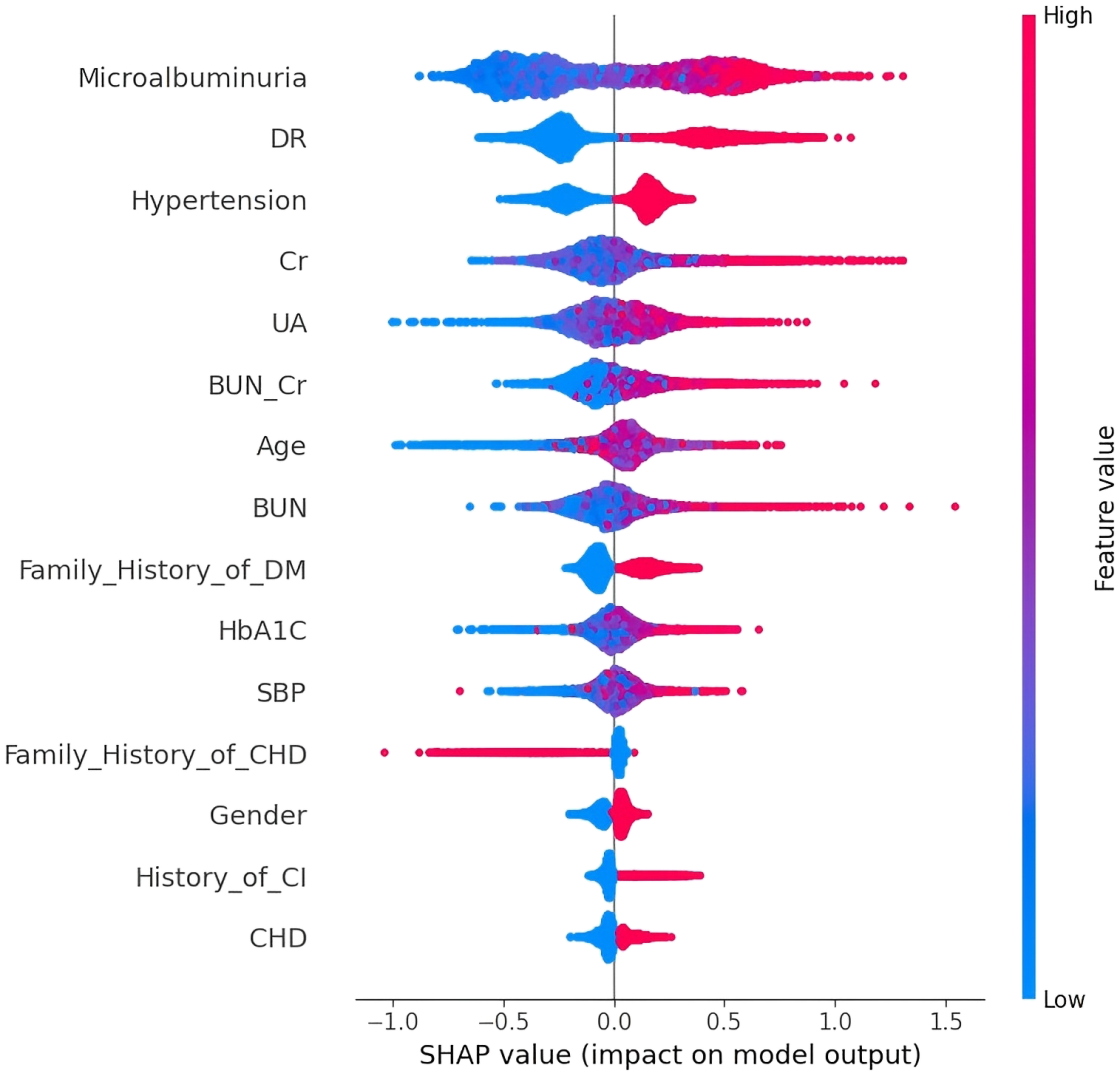
SHAP analysis of XGBoost model. A visual representation of each feature in the XGBoost model shows the relationship between the importance of each feature. The color represents the value of the variable, with red representing a larger value and blue representing a smaller value.

**Figure 6:**
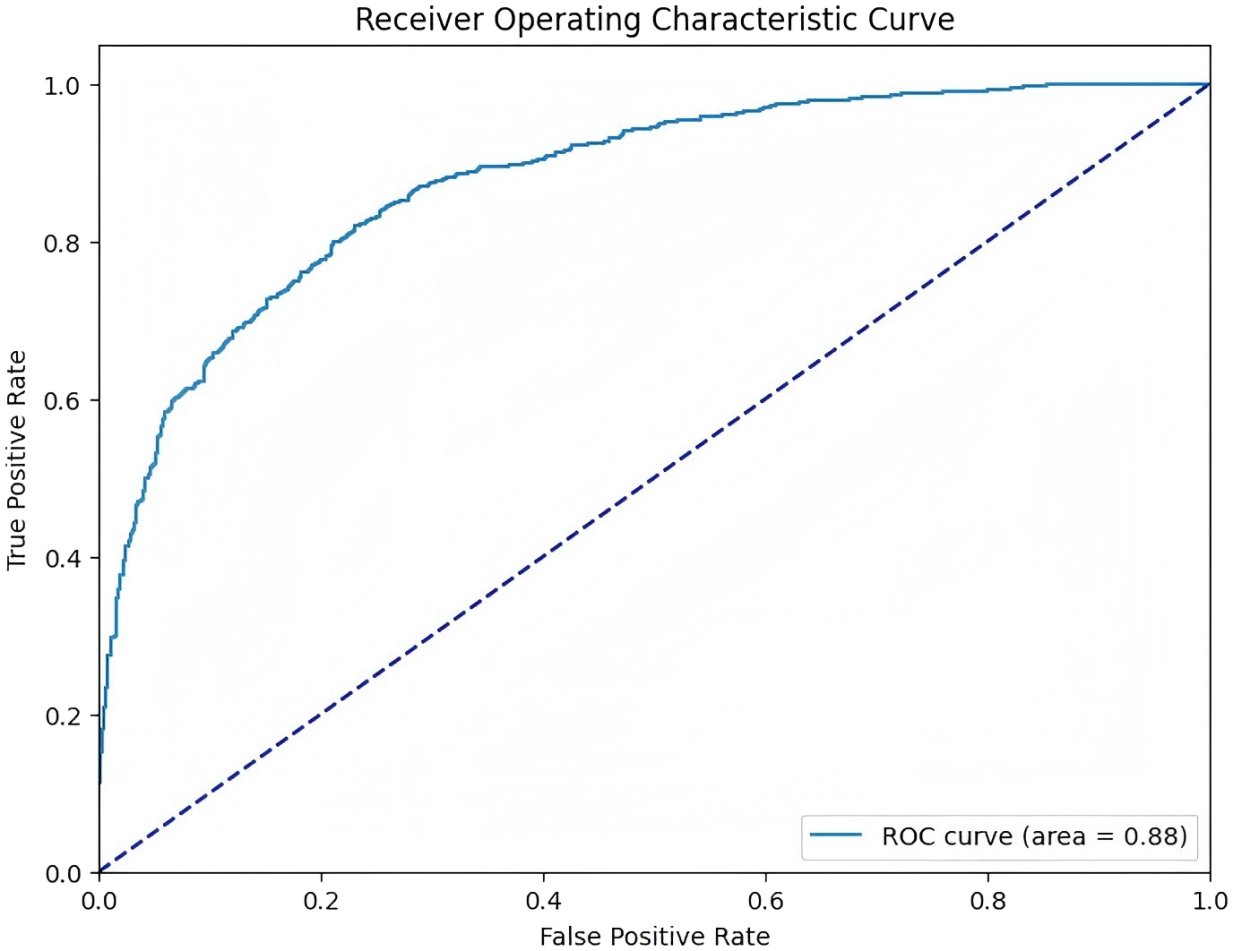
External validation ROC curve.

Furthermore, after selecting the XGBoost model, the SHAP package was used to analyze the XGBoost model, which reflects the influence of each feature in the sample and shows the positive and negative influences (**Figure 5**). For the external validation dataset, data of 1,508 patients were collected to validate the performance of the established XGBoost model (AUC = 0.878, 95% CI (0.920-0.939), accuracy = 0.788, sensitivity = 0.783, specificity = 0.793, F1 score = 0.791) (**Figure 6**).

#### Decision curve analysis

**Figure 7:**
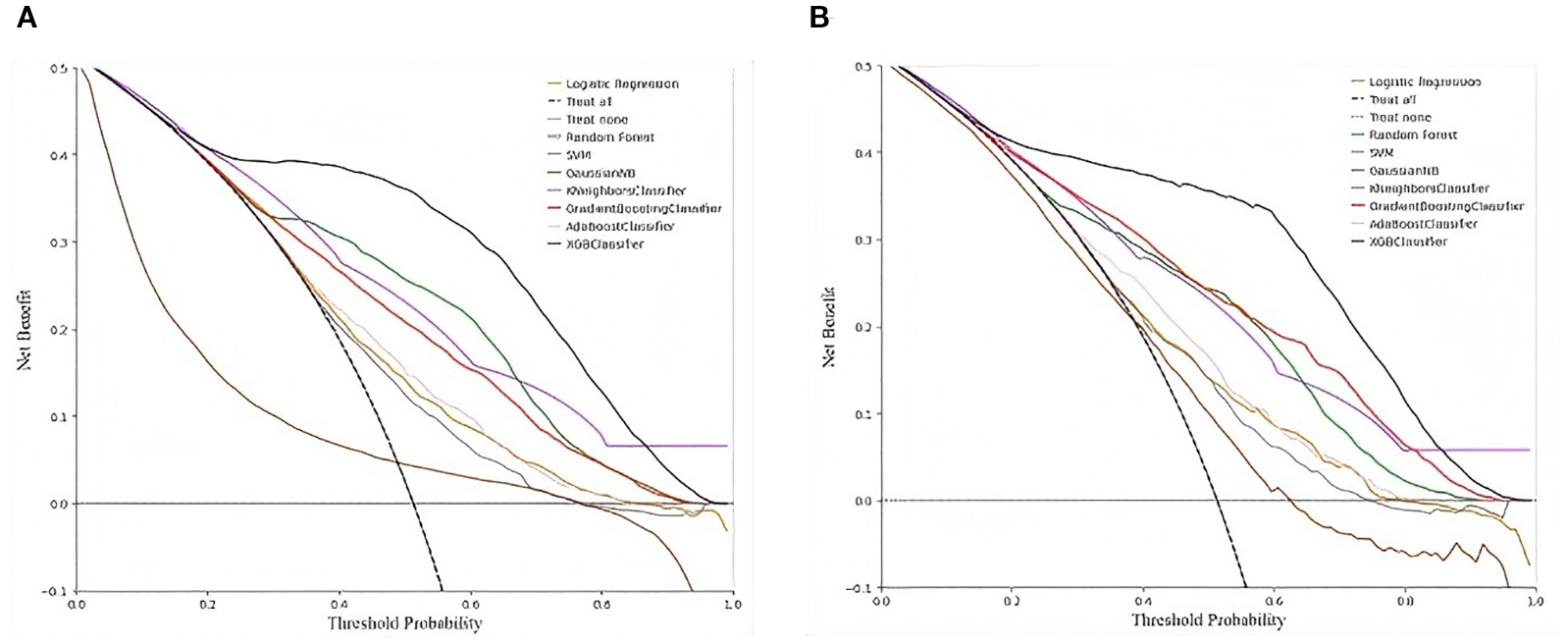
Decision curve analysis of the eight models predicting the incidence of DKD. **(A)** Comparison of DCA among the eight machine learning models in the training set, **(B)** Comparison of DCA among the eight machine learning models in the validation set.

To further investigate the clinical application of the XGBoost model, a comparison of the DCA among the eight machine-learning models was conducted. The results still show a larger net benefit across a range of threshold probabilities in the XGBoost model (**Figure 7**). For application of the XGBoost model, the best cut-off for the prediction probability of the proposed model was 50.7%. If the model predicted a probability > 50.7%, the risk of developing DKD in patients with T2DM was higher (**Table 2**).

### Application of the model

**Figure 8:**
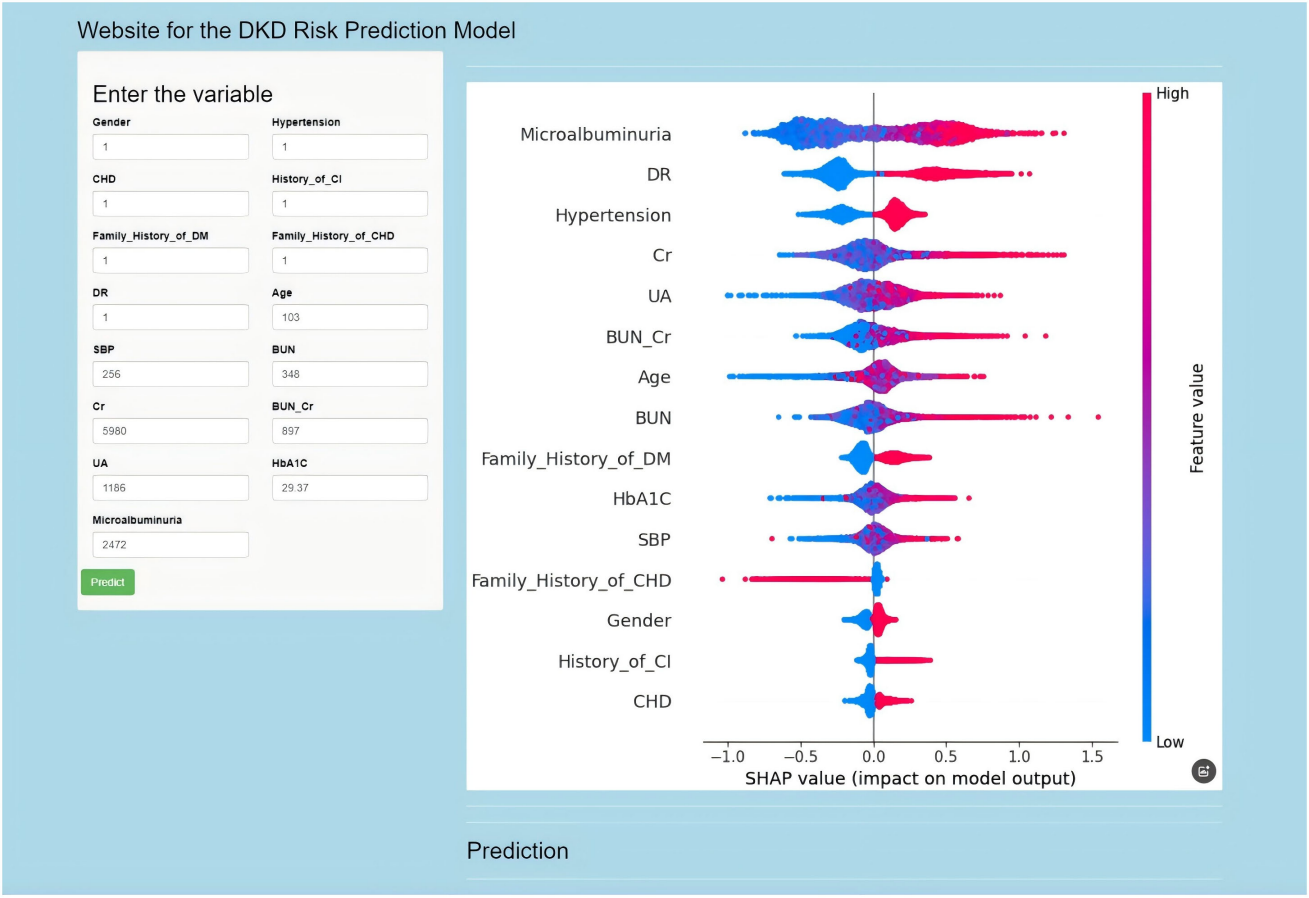
Establish a website predictor for the risk of developing DKD based on the XGBoost model. The URL provided is: https://liting3659078.shinyapps.io/myrapp/.

Last, based on a cut-off value of 50.7% in this model, we constructed an online prediction calculator for DKD risk (https://liting3659078.shinyapps.io/myrapp/, **Figure 8**), by which a practice of two representative patients exhibited a good predictive effectiveness (**Supplementary Figure 1**). The indicators related to these two patients are shown in **Supplementary Table 2**.

## Discussion

In China, the management of DKD in patients with T2DM faces challenges characterized by low screening rates, low awareness among patients, low treatment rates, unattainable therapeutic goals, and insufficient community-based preventive capacities. Chen et al. (15) conducted a 7-year follow-up study on 907 diabetic patients from the Taopu Community Health Service Center in Putuo district of Shanghai, revealing that by 2015, the screening rate of DKD was merely 55.1%, which is notably lower than that of diabetic neuropathy and retinopathy (77.6%). Hence, developing strategies to efficiently increase the screening rate among high-risk populations and implementing clinical prediction tools could be a solution.

The present study was the first to ensure the 15 predictive variables affecting the occurrence of DKD in patients with T2DM as follows: gender, age, SBP, BUN, Cr, BUN/Cr, UA, HbA_1c_, microalbuminuria, presence of DR, hypertension, CHD, history of cerebral infarction, family history of diabetes, and family history of CHD following LASSO regression analysis, which can balance optimal fitting error and adjust the quantity and magnitude of model parameters, thereby identifying those features with enhanced predictive power over the outcome variable. This process reduces the model complexity, mitigates multicollinearity, prevents overfitting, and ultimately enhances the generalizability of the model. We constructed and compared the predictive efficacy of eight machine learning models for forecasting the DKD aspect, and the XGBoost model exhibited superior predictive capabilities in both the training and validation sets, with AUC values of 0.932 and 0.930, and F1 scores of 0.847 and 0.848, respectively. Moreover, this optimal model had a larger net benefit and threshold probability, demonstrating the clinical significance of DKD management.

The 15 predictive variables related to the occurrence of DKD in patients with T2DM were ranked as follows: microalbuminuria, presence of DR, hypertension, Cr, UA, BUN/Cr, age, BUN, family history of diabetes, HbA_1c_, SBP, family history of CHD, sex, history of cerebral infarction, and presence of CHD. Microalbuminuria was found to have the most significant effect on the occurrence of DKD. This is likely because microalbuminuria is a crucial biomarker in the early stages of DKD. When the kidneys of diabetic patients begin to sustain damage, microalbumin begins to appear in the urine, acting as an early indicator of renal impairment. A systematic review has indicated that DR is closely associated with nephropathy. The presence of DR increases the risk of nephropathy and serves as a predictive indicator of microalbuminuria progression (16). Hypertension is a major risk factor for the progression of DKD and the occurrence of cardiovascular diseases and death, and persistent hypertension exacerbates the burden on the kidneys (17–19). UA, Cr, BUN, and microalbumin are common indicators of renal function, with Cr, BUN, and UA playing essential roles in early DKD screening (20). The results of our study were similar to the results of Li et al. (21) by multifactorial logistic regression analysis, and the prevalence of DKD was significantly higher in patients with T2DM aged ≥50 years [*OR* = 4.011, 95%CI (3.152-5.104)], which is consistent with the results of our study. As we known that, HbA_1c_ serves as a pivotal index for evaluating long-term glycemic control in diabetic patients, and Ali et al. (22) showed that HbA_1c_ plays a significant role in the development of DKD, with an association between HbA_1c_ and microalbuminuria. Microalbuminuria is a crucial early marker of diabetic nephropathy, and when renal damage begins in diabetic patients, microalbuminuria appears in the urine. Elevated HbA_1c_ levels often correlate with increased microalbuminuria. In our study, HbA_1c_ emerged as the most influential risk factor for DKD occurrence, likely because all participants were patients with type 2 diabetes and HbA_1c_ was a key indicator selected by LASSO regression. In this study, sex influenced the occurrence of DKD, with males at a higher risk. Research shows that sex differences play a key role in the progression of DKD in T2DM patients, as the DKD incidence rate in males (23.2%) is higher than that in females (19.8%) (8). Logistic regression analysis revealed that a family history of diabetes was significantly associated with the development of DKD (*P* < 0.05) (23).

Using the XGBoost model established based on the above characteristic variables, we conducted an external validation on a dataset that was not used for training and testing. The results showed that relatively excellent AUC, F1 score, and so on were obtained. Thus, with the advent of the artificial intelligence era, a growing body of research has shown that many models have been developed to predict the occurrence and prognosis of diseases, even the early identification of high-risk populations for DKD. However, a comprehensive comparison of multi-predictive models on performance and clinical value as well as online application remains unknown. Additionally, previous studies required manual calculations with model inputs, which significantly limited their practicality. To enhance the usability of the constructed models, we designed and deployed an online prediction calculator hosted to facilitate its availability to clinicians and patients and explored one example confirming its practical application efficiency.

This study has several limitations attention as follows: 1) The information on patients’ medication use wasn’t included in this study, preventing the identification of specific drugs and their combinations’ impact on the development of DKD. 2) The data were from hospital settings excluding community-dwelling T2DM populations, which account for a large number of high-risk DKD patients.

Overall, our study provides an optimal predictive model (XGBoost model) integrated with 15 featured indicators on a dedicated website for DKD occurrence in T2DM patients. This tool can effectively support clinical decision making and patient guidance.

## Data Availability

The raw data supporting the conclusions of this article will be made available by the authors, without undue reservation.

## Supplementary material

Supplementary Table 1 Variable assignment.

Supplementary Table 2 Indicators related to DKD and non-DKD patients.

Supplementary Figure 1 DKD online predictor for running results of two patients. **(a)** The predicted risk of developing DKD in Case 1 was 43.08% (< 50.7%), and **(b)** the predicted risk of developing DKD in Case 2 was 51.90%(> 50.7%).
