# Supplementary material for: A machine learning model for predicting the risk of diabetic nephropathy in individuals with type 2 diabetes mellitus
Source: Front Endocrinol (Lausanne). 2025 Oct 15;16:1587932. doi: 10.3389/fendo.2025.1587932 (PMC12568428; doi:10.3389/fendo.2025.1587932)
Supplement: Supplementary Table 1 — Variable assignment. [file DataSheet1.docx]

**Supplementary Table 1 Variable assignment**

| Variable | Assignment |
| --- | --- |
| Gender | 0=male，1=female |
| Age | Measured Value |
| BMI | Measured Value |
| SBP | Measured Value |
| DBP | Measured Value |
| Smoking | 0=no，1=yes |
| Drinking | 0=no，1=yes |
| FBG | Measured Value |
| hs-CRP | Measured Value |
| WBC | Measured Value |
| LYM | Measured Value |
| NEUT | Measured Value |
| MONO | Measured Value |
| PLT | Measured Value |
| PDW | Measured Value |
| P-LCR | Measured Value |
| D-dimer | Measured Value |
| BUN | Measured Value |
| Cr | Measured Value |
| BUN/Cr | Measured Value |
| UA | Measured Value |
| GLU | Measured Value |
| HbA_1c_ | Measured Value |
| TG | Measured Value |
| TC | Measured Value |
| HDL | Measured Value |
| LDL | Measured Value |
| APOA1/APOB | Measured Value |
| AST | Measured Value |
| ALT | Measured Value |
| DBIL | Measured Value |
| IBIL | Measured Value |
| Alb | Measured Value |
| α1-MG | Measured Value |
| β2-MG | Measured Value |
| Microalbuminuria | Measured Value |
| DR | 0=no，1=yes |
| Hypertension | 0=no，1=yes |
| CHD | 0=no，1=yes |
| Cerebral infarction | 0=no，1=yes |
| Hypokalemia | 0=no，1=yes |
| Hyperlipidemia | 0=no，1=yes |
| History of coronary heart disease | 0=no，1=yes |
| History of cerebral infarction | 0=no，1=yes |
| Family history of hypertension | 0=no，1=yes |
| Family history of diabetes | 0=no，1=yes |
| Family history of CHD | 0=no，1=yes |

**Supplementary Table 2 Indicators related to DKD and non-DKD patients**

| Variable | Case 1 | Case 2 |
| --- | --- | --- |
| DKD | 0 | 1 |
| Gender | male | male |
| Age, years | 82 | 19 |
| SBP, mmHg | 125 | 135 |
| BUN, mmol/L | 5.5 | 7.0 |
| Cr, μmol/L | 65 | 71 |
| BUN/Cr | 84.61 | 98.59 |
| UA, μmol/L | 182 | 317 |
| HbA_1c_, % | 13.5 | 8.5 |
| Microalbuminuria, g/L | 25.4 | 263.15 |
| DR | 0 | 0 |
| Hypertension | 0 | 0 |
| CHD | 0 | 0 |
| History of cerebral infarction | 1 | 0 |
| Family history of diabetes | 0 | 1 |
| Family history of CHD | 1 | 0 |

a

b


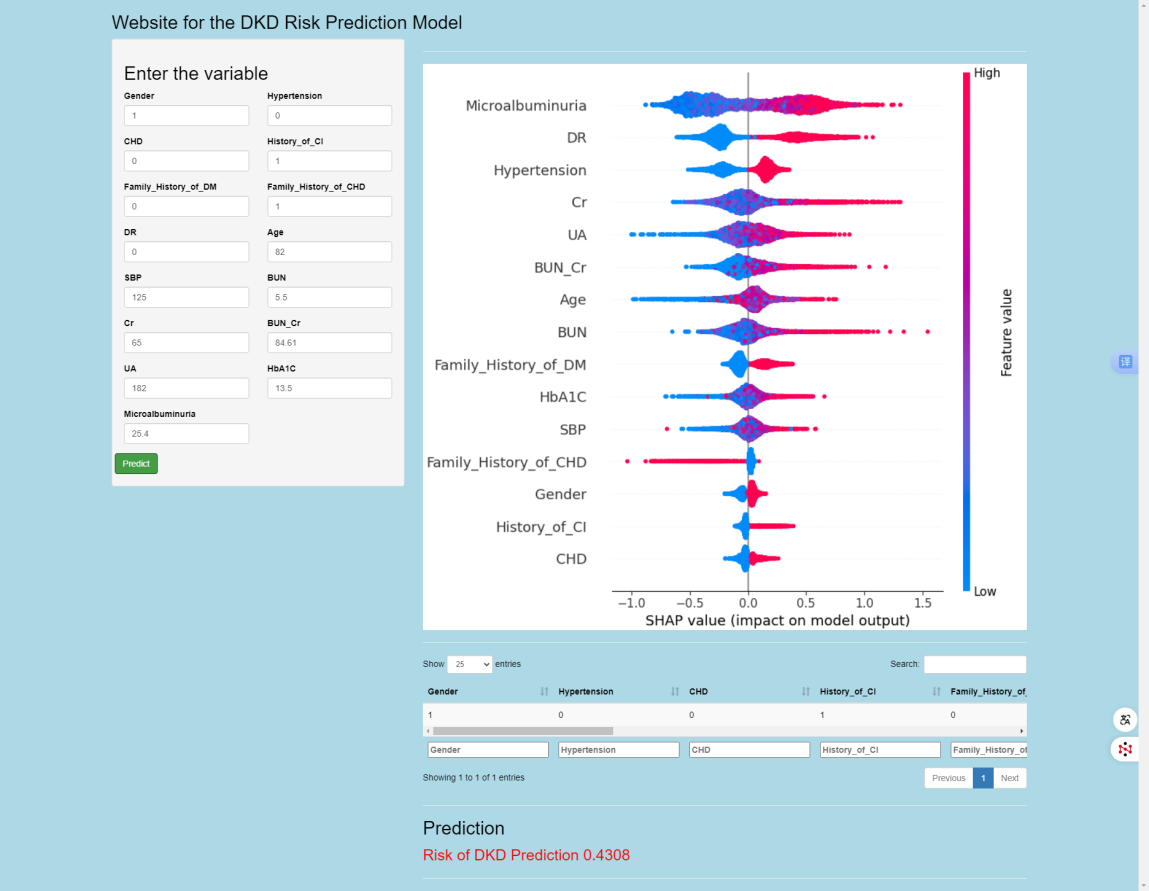

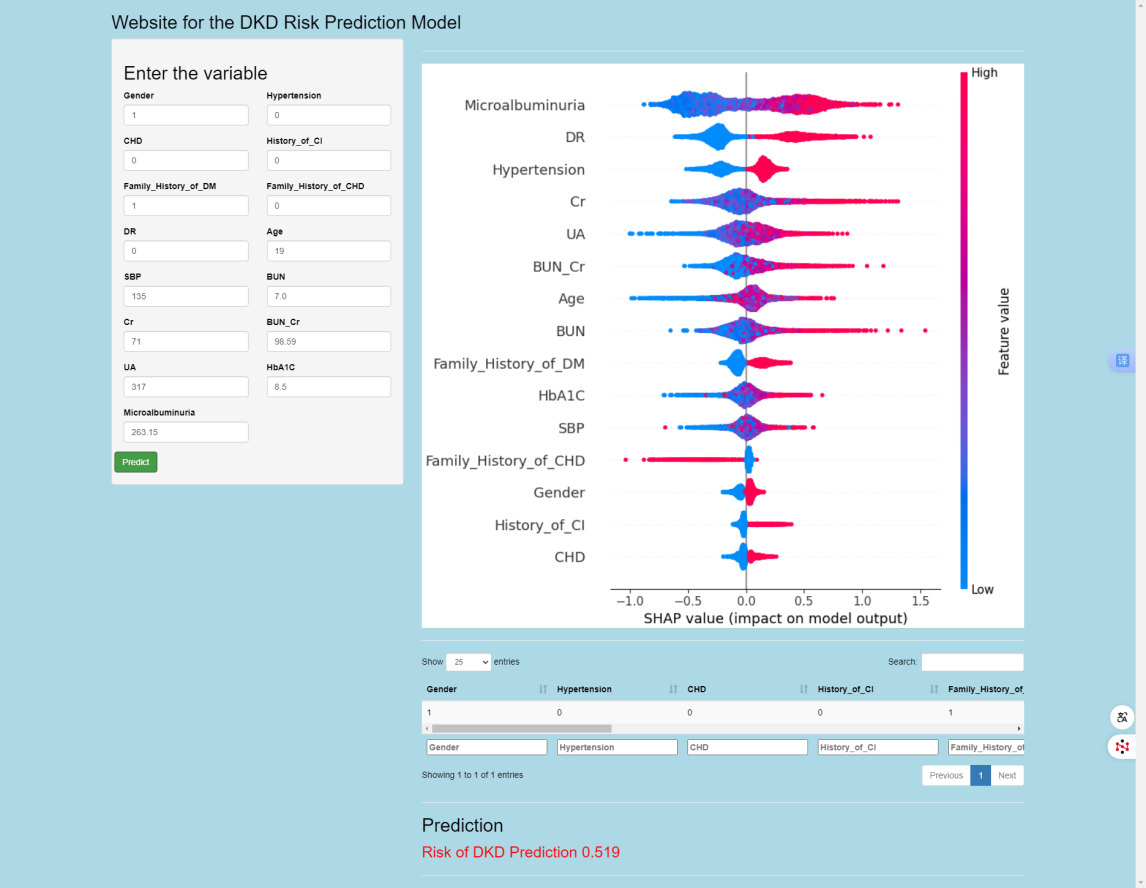


**Supplementary Figure 1 DKD online predictor for two patients running results.** (a) The predicted risk of developing DKD for Case 1 is 43.08% (< 50.7%), (b) The predicted risk of developing DKD for Case 2 is 51.90%(> 50.7%).
